# Exploring the Emotional Dysregulation Scale-Short Form in isolated, confined, and extreme environments

**DOI:** 10.4102/ajopa.v5i0.119

**Published:** 2023-03-22

**Authors:** Charles H. van Wijk

**Affiliations:** 1 Department of Global Health, Faculty of Medicine and Health Sciences, Stellenbosch University, Cape Town, South Africa; 2 Institute for Maritime Medicine, Simon’s Town, South Africa

**Keywords:** EDS-S, emotional dysregulation, Isolated, confined and extreme environments, mental health, South Africa, validity

## Abstract

The Emotional Dysregulation Scale-Short Form (EDS-S) may have potential for assessing emotional dysregulation (ED) both in general clinical mental health environments and in specialised work settings. Before it can be used fairly and appropriately, evidence of its validity in the local South African (SA) context is required. This study thus explored its psychometric characteristics among local working adult samples by pursuing three specific objectives, namely, to investigate its structural validity, its construct validity, and issues around practical use (e.g. priming bias and ability to predict performance). Data were collected across four samples that comprised general workers and specialised naval personnel (total *N* = 1374), who also completed measures of clinical mental health and other adjustment difficulties. Statistical analysis included examination of socio-demographic effects, internal consistencies, confirmatory factor analysis, measurement invariance, and associations with measures of mental health and adjustment difficulties (including binomial logistic regressions and receiver operating/operator characteristics curve analyses). This study reported evidence of structural and criterion validity, with significant associations to measures of mental health and adjustment difficulties, for the 12-item EDS-S in non-clinical samples of SA workers. The study further provided preliminary support for its predictive utility in specialised work environments. Preliminary evidence of validity of the EDS-S in SA worker samples with sufficient English proficiency was demonstrated.

**Contribution:**

There is some support for the use of the EDS-S in clinical research and applied practise. However, caution must be observed for possible effects of language proficiency and further research into the role of language is required.

## Introduction

### Overview

Isolated, confined and extreme (ICE) environments pose special challenges to psychological performance, and optimal adaptation in such environments is required to ensure well-being. Successful adaptation is contingent on, among other things, appropriate emotional regulation. Various mechanisms exist to measure emotional regulation, and this article investigates validity aspects of one such tool, namely, the Emotional Dysregulation Scale (EDS).

### Isolated, confined and extreme environments

Isolated, confined and extreme environments refer to settings characterised by hostile external conditions, and an exposure to a range of context-specific physical, mental and social stressors, and often require engineering technology to maintain human survival. Isolated, confined and extreme environments are, for instance, underwater habitats, spacecraft, remote weather stations, polar outposts, and in certain circumstances, ships at sea (Suedfeld & Steel, 2000; Van Wijk & Martin, 2021).

Such ICE environments may present considerable and often unique configurations of psychological challenges to individuals and groups working in such settings. Challenges to survival may include a hostile climate and the mastery of specialised equipment for life support, as well as demands of constant vigilance – where neither critical nor routine tasks can be avoided or postponed, and where mistakes may have severe consequences. Social challenges include restricted communication with the outside world, cramped living spaces, enforced intimacy with individuals not of one’s choosing (Sandal, 2000, p. A37), navigating evolving group dynamics and emotional isolation.

Reports describe how persons in ICE environments are exposed to exceptionally high levels of stress, resulting in higher-than-average rates of somatic symptoms, anxiety, depression, hostility, and mild cognitive impairment. These symptoms of stress appear to manifest themselves as health problems, reduced emotional well-being, decreased performance, and interpersonal tension (Basner et al., 2014; Kanas et al., 2009; Palinkas & Suedfeld, 2008; Rohrer, 1961; Sandal, 2000; Shea et al., 2009). Liu et al. (2016) further demonstrated that isolation and confinement result in a decreased ability to regulate emotions, as well as an increased vulnerability to negative emotions.

Appropriate psychological adaptation to these challenges would be critical to achieve and maintain both optimal performance and optimal well-being in such settings. Broadly stated, psychological adaptation refers to an individual’s ability to adjust to changes in their environment to optimise personal functioning.

In ICE environments, successful psychological adaptation is traditionally operationalised in terms of Gunderson’s Antarctic Triarchy (Gunderson, 1973; Palinkas et al., 2000; Suedfeld & Steel, 2000), which reflects three domains, namely:

- Task ability (referring to the quality of work output).
- Sociability (referring to the quality of interpersonal interaction; sometimes referred to as ‘social compatibility’).
- Emotional stability (referring to the quality of internal self-regulation).

Psychological adaptation to ICE environments is of increasing interest to southern Africa. For example, the South African (SA) government maintains a polar icebreaker and three research/weather stations in Antarctica and Islands as part of the South African National Antarctic Programme (https://www.sanap.ac.za/). The South African Navy (SAN) operates long-range patrol vessels (e.g. frigates) and submarines (https://en.wikipedia.org/wiki/South_African_Navy), and a number of private companies in the oil and gas industry operate offshore drilling platforms from the Angolan to the Mozambican coasts. All of these examples may qualify as isolated and confined environments, and while not all are necessarily extreme, they are certainly unusual for those accustomed to living on terra firma.

### Emotional dysregulation

The emotional regulation (and dysregulation) aspect of adaptation is of particular interest, as it underpins personal performance across many facets of daily life, including family, work and sport (Gross & Thompson, 2007). In ICE environments, individuals with more adaptive emotional regulation would be expected to more effectively manage their personal performance across work output, social interactions, and affective states, especially under the psychological rigorous demands of ICE environments (Palinkas & Suedfeld, 2008). In contrast, individuals with less adaptive emotional regulation might be expected to have greater difficulty managing their personal performance across the same three domains. Emotional regulation thus acts across domains to influence the maintenance of quality work output, social relations and emotional well-being. It may therefore be useful to know of problematic emotional regulation in individuals, as this can prime programme managers to either better prepare individuals for the rigours of ICE environments or to advise against such exposures.

Emotional regulation can be defined as the ability of an individual to correctly identify, monitor, express and modulate the intensity and duration of an emotion or set of emotions (American Psychological Association [APA], 2022a; Cole et al., 1994; Raimondi et al., 2022). Emotional dysregulation (ED) refers to the difficulty or inability to carry out this process, and in particular refers to extreme or inappropriate emotional response to a situation (APA, 2022b). As such, ED reflects deficits in awareness and acceptance of emotions, as well as in regulation strategies to manage intense, negative and shifting emotional states (Gross & Thompson, 2007; Powers et al., 2015). Emotional dysregulation is currently understood as a trans-diagnostic construct that has an impact on many psychological conditions, spanning from, among others, mood and anxiety disorders, substance use and personality disorders to autism spectrum disorder, psychological trauma and brain injury (cf. APA, 2022b; Powers et al., 2015; Raimondi et al., 2022; for summaries).

Developmental research suggests that these self-regulatory deficits emerge from an interaction of intrinsic temperamental and biological factors, as well as extrinsic intrusions, such as exposure to traumatic experiences, particularly in early life (Bradley et al., 2011; Powers et al., 2015).

Emotional dysregulation is not the same as negative affect. Generally speaking, negative affect reflects the types of emotions people have (e.g. anger, fear and sadness), while emotion regulation reflects the ability to adaptively manage the intensity and duration of emotions (including negative ones) as they arise (Powers et al., 2015, p. 86). This distinction has an important practical application, in that patients can be taught strategies for how to manage intense, negative emotions as they occur (Powers et al., 2015). Components of ED include a tendency for emotions to spiral out of control, change rapidly, get expressed in intense and unmodified forms, and/or overwhelm both coping capacity and reasoning (Bradley et al., 2011).

### Measuring emotional dysregulation

A number of self-report instruments are available to measure ED, including the 36-item Difficulties in Emotion Regulation Scale (DERS; Gratz & Roemer, 2004), which measures six dimensions of difficulties in emotion regulation, and the 10-item Emotional Regulation Questionnaire (ERQ; Gross & John, 2003), which measures two emotional regulation strategies. In spite of their widespread use, they also have serious limitations (cf. Powers et al., 2015; Raimondi et al., 2022, for critique).

Bradley et al. (2011) developed the 24-item EDS. Items are scored on a seven-point Likert scale and assess domains of emotional experiencing, cognition and behaviour. The scale demonstrated high internal consistency, replicated across samples (Chanana & Sharma, 2019). Emotional Dysregulation Scale-24 scores were significantly correlated to childhood trauma and negative affect, as well as significant predictors of post-traumatic stress symptoms, history of alcohol and drug abuse problems, depressive symptoms and lower global adaptive functioning (Bradley et al., 2011). While EDS-24 scores were significantly associated with all the subscales of the DERS, it also demonstrated incremental validity over the DERS in predicting different psychopathological conditions. Recent studies have supported the use of the EDS in a variety of clinical populations, for example, with patients suffering from mood disorders or post-traumatic stress disorder (PTSD; Christ et al., 2019; Mekawi et al., 2020; Pencea et al., 2020).

In response to the criticisms of the DERS, ERQ and length of the EDS-24, Powers et al. (2015) developed the EDS-short form (EDS-S), based on an exploratory factor analysis (EFA) of the original EDS 24-item scale, which yielded one factor. The 12 items with the highest loadings were then chosen for the EDS-S. The bivariate correlation between the 24-item and 12-item EDS scales was extremely high (*r* = 0.98, *p* < 0.001). The EDS-S retained the seven-point Likert scale, with items assessing the domains of emotional experiencing (‘emotions overwhelm me’), cognition (‘when I’m upset, everything feels like a disaster’), and behaviour (‘when my emotions are strong, I often make bad decisions’) and higher scores indicating higher ED. High internal consistency for the EDS-S has been reported (Mandavia et al., 2016; Michopoulos et al., 2015; Powers et al., 2015; Raimondi et al., 2022). The EDS-S demonstrated a significant correlation with DERS, and appeared predictive of depressive symptoms, PTSD symptoms, alcohol abuse, borderline personality disorder, general psychopathology, suicidality and psychiatric hospitalisation, and was negatively associated with positive affect and resilient coping (Mandavia et al., 2016; Michopoulos et al., 2015; Powers et al., 2015; Raimondi et al., 2022). Confirmatory factor analysis (CFA) of an Italian version suggested a unidimensional structure (Raimondi et al., 2022). Table 1 provides a summary of published data on the EDS-24 and EDS-S.

**TABLE 1:** Summary of published data on the Emotional Dysregulation Scale.

Study reference	α	Mean	s.d.	Dimensionality
**24-item EDS**				
Bradley et al. (2011)	0.97	-	-	-
Chanana and Sharma (2019)	0.93	95.20	28.27	-
Powers et al. (2015)				EFA = 1 factor, explaining 54% of the variance
**12-item EDS-S**				
Powers et al. (2015)	-	-	-	-
Sample 1	0.93	33.3	18.2	-
Sample 2	0.94	38.9	21.7	-
Sample 3	0.95	38.1	21.7	-
Michopoulos et al. (2015)	0.90	-	-	-
Mandavia et al. (2016)	0.95	39.97	21.92	-
Pencea et al. (2020)	0.95	38.2	22.0	-
Raimondi et al. (2022)	0.94	37.9	15.7	CFA = unidimensional structure

s.d., standard deviation; EFA, exploratory factor analysis; CFA, confirmatory factor analysis; α, Cronbach alpha coefficient; EDS, Emotional Dysregulation Scale; EDS-S, Emotional Dysregulation Scale-Short Form.

### Aims and overview of studies

The EDS may have potential for assessing ED in both specific ICE environments and clinical mental health settings. For example, responses to the EDS could be used to guide decisions around inclusion/exclusion of individuals applying for missions in ICE environments, or to guide appropriate preparation or advance intervention for such persons. It could also be used for research within clinical settings to better understand the role of emotional regulation in the development of, or protection against, mental disorders. However, neither its fair and unbiased use (Employment Equity Act, 1998) nor its clinical or practical validity (i.e. accuracy in identifying risk) have been established in the SA context. Validation is a constant process, involving a continuum of evidentiary support, including evidence of internal structures and effects of context and sample characteristics (Schaap & Kekana, 2016). Therefore, before it can be used with confidence, a better understanding of the instrument in the indigenous SA context is required.

This study thus set out to explore the psychometric characteristics of the scale among local population samples. It used data collected across four studies to pursue three specific objectives: Firstly, it investigated the structural validity of the EDS. Secondly, it investigated the construct validity of the EDS, by exploring its associations with measures of common mental disorders and indicators of adjustment difficulties, as well as describing the EDS profile in a group that has demonstrated good adaptation in an ICE environment. Thirdly, it investigated two issues around practical use, namely the EDS’ susceptibility to priming bias and the EDS’ ability to predict self-rated performance in ICE contexts.

The studies were set up as follows: In general terms, validity refers to the extent to which a scale measures what it claims to measure. Study 1 thus investigated, firstly, the structural validity of the EDS by describing its psychometric characteristics in a general SA workplace sample (including internal consistency and test–retest reliability, socio-demographic effects and dimensionality), and secondly, the construct validity of the EDS by exploring its association with measures of common mental disorders and other indicators of mental (ill)health history, work adjustment and experience of stress overload.

Priming is the phenomenon according to which the recent experience of a stimulus facilitates or inhibits later processing of the same or a similar stimulus (APA, 2022c). In other words, it describes how the introduction of one stimulus influences how people respond to a subsequent stimulus (Cherry, 2021). One example is repetition priming, in which the presentation of a particular stimulus increases the likelihood that participants will identify the same or a similar stimulus later in a test. In semantic priming, presentation of a word or symbol influences the way in which participants interpret a subsequent word or symbol (APA, 2022c). Priming works by activating an association or representation in memory, and can work with stimuli that are perceptually, linguistically or conceptually related (Cherry, 2021). This phenomenon is thought to generally occur outside of conscious awareness. In research, measures of ED are often used in combination with measures of other psychological constructs, raising the question how such combinations may influence responses on the EDS, depending on where – in a sequence of measures – the EDS is administered. Study 2 thus aimed to investigate the EDS’ susceptibility to priming bias by exploring the effects of mood state responses on the priming of EDS responses.

Successful psychological adaptation in ICE environments has traditionally been operationalised in terms of Gunderson’s Antarctic Triarchy, namely performance in the domains of task ability (work-output), sociability (interpersonal relations) and emotional states. By monitoring and modulating (APA, 2022a) a person’s inner state, adaptive emotional regulation may act across these three domains to optimise personal performance (referring here to work and social behaviour, well-being, etc.). Study 3 thus aimed to investigate the EDS’ ability to predict performance in ICE contexts by exploring EDS-S total score associations with firstly, self-rated performance assessment on the Antarctic Triarchy, and secondly, mood state as measured by the Brunel Mood Scale (BRUMS), at the end of a 3-month ICE mission.

Submarines constitute a very specific example of an ICE environment, and submariners have traditionally been considered as particularly good adaptors (Kimhi, 2011; Van Wijk, 2017, 2022; Weybrew & Noddin, 1979). Good emotional regulation – and low EDS-S scores – would be expected from this group. Study 4 thus aimed to describe the EDS-S profile of a population that has demonstrated good adaptation in an ICE environment, namely a small sample of SAN submariners.

All four studies used a cross-sectional survey design. It needs to be noted that the EDS and other measuring scales employed in this study were designed for mental health screening and not for clinical diagnosis purposes, and their use for diagnostic ends are not recommended.

## Methods

### Study 1

#### Participants and procedure

The sample was drawn from non-clinical and skilled worker populations who volunteered to complete the scales and questionnaires during employer-sponsored occupational health surveillance initiatives. Prior to giving their consent and providing any information, volunteers were briefed that the completion of the EDS-24 would not influence their health screening or any subsequent health support.

The mean age of the 1006 participants was 33 years (±8, range: 20–60 years), and 33.4% of the sample were women. English as the first language was spoken by 19.1% of the sample, with the rest reporting the other 10 SA official languages as their mother tongues. All participants self-reported as proficient in English; however, actual English proficiency was not objectively established. The detailed distribution across language and occupational fields is presented in Table 2.

**TABLE 2:** Sample distribution across home language and occupational field.

Demographic distribution	*n*	%
**Language**		
English	192	19.1
Afrikaans	159	15.8
IsiXhosa	137	13.6
Setswana	125	12.4
Sesotho	107	10.6
IsiZulu	104	10.3
Sepedi	83	8.3
Tshivenda	48	4.8
Tsonga	25	2.5
SiSwati	15	1.5
Ndebele	8	0.8
Unknown	3	0.3
**Occupational sectors**		
Administrative/clerical	149	14.8
Technical/engineering	235	23.4
Catering/hospitality	131	13.0
Security sector	280	27.8
Other	211	21.0

#### Measures

**Emotional Dysregulation Scale:** The full 24-item EDS (Bradley et al., 2011) was administered in its standard format, in English. Respondents were asked to rate each item on a seven-point Likert scale (1 = *‘Not true’*, 7 = *‘Very true’*), with higher scores reflecting greater ED.

A subsample (*N* = 131) competed the same version of the EDS 35 days after the first administration. This was a purely convenience sample (i.e. who could be contacted and was available at the time), used to investigate test–retest reliability.

**Self-report questionnaire:** Participants also completed a self-report questionnaire with four sections: mental health history, consisting of three items with *yes/no* answers, enquired about previous admission to hospital or clinic for mental health concerns, previous psychological or psychiatric out-patient treatment, and previous treatment for alcohol or substance abuse or addiction. Adjustment at work, consisting of two items with *yes/no* answers, enquired about concerns regarding interpersonal relations in their workgroup (conflict with co-workers, supervisors), and disciplinary issues at work during the past 2 years. Domestic discord enquired about difficulties in relationships with partner/immediate family. Finally, two items reflected the very brief screen for adult Attention Deficit/Hyperactivity Disorder (ADHD) and scores ≥ 1 were considered suggestive of ADHD (Van Wijk & Firfirey, 2020; Zimmerman et al., 2017).

**Indicators of common mental disorders:** Current clinical syndromes were identified using locally validated (cf. Van Wijk et al., 2021) scales for common mental disorders: The Patient Health Questionnaire for depression (PHQ-9; Gilbody et al., 2007) was used to screen for depression, with a score of ≥ 10 used for identifying cases (α = 0.79 for this study). The Generalised Anxiety Disorder scale (GAD-7; Löwe et al., 2008) was used to screen for generalised anxiety disorder, with a score of ≥ 10 identifying cases (α = 0.82 for this study). The primary care screen for PTSD using DSM-5 criteria (PC-PTSD-5; Bovin et al., 2021) was used to screen for PTSD, with a score of ≥ 3 identifying cases (α = 0.73 for this study), and the CAGE (cut, annoyed, guilty, eye-opener) questionnaire (Dhalla & Kopec, 2007) was used to screen for problematic alcohol use, with a score of ≥ 2 identifying cases (α = 0.57 for this study).

**Stress overload:** Current stress overload, in a subsample of 224 participants, was measured with the 10-item Stress Overload Scale-Short Form (SOS-S; Amirkhan, 2018). This was to identify participants who were experiencing the demands of life as overwhelming their available resources. Previous SA research suggested that scores > 20 were associated with significant mental health difficulties (Van Wijk, 2021).

#### Data analysis

All statistical analyses were conducted by means of Statistical Package for Social Sciences (SPSS) (IBM SPSS for Windows, version 27) and analysis of moment structures (AMOS). Means, standard deviations (s.d.), and range (for the full scale and the short form) were calculated.

To confirm the 12-item EDS-S, the current study carefully replicated the original process undertaken by Powers et al. (2015), which included an EFA and retaining items with the highest loadings. The rest of the analysis is based on the 12-item EDS-S.

The effects of socio-demographic variables were explored using Pearson’s correlation coefficients and analysis of variance (ANOVA) for age, as well as *t*-tests for independent samples for gender and language effects. For this analysis, language was coded into two groups, namely English first language (19.1%) and non-English first language (80.8%), and age was coded into four groups (20–29, 30–39, 40–49 and 50–60).

Internal consistencies were examined with Cronbach’s α, inter-item correlations and corrected item-total correlations. Test–retest reliability was examined by comparing the two administrations of the EDS, 35 days apart (*N* = 131), using a paired sample *t*-test.

The earlier EFA with the 24-item version suggested a single factor, and a CFA previously found a unidimensional structure in an Italian version of the EDS-S (Raimondi et al., 2022). A CFA was thus conducted to test a model with a unidimensional structure. Confirmatory factor analysis is used to test whether the data fit a hypothesised measurement model (Marker, 2002). The Maximum Likelihood estimator was used to explore a one-factor model fit. For a CFA, the global fit χ^*2*^ would ideally be small and not significant, but it is rarely achieved, and the following indices with cut points were also taken into consideration: the root mean square error of approximation (RMSEA) should be < 0.06 to < 0.08 for continuous data, while both the comparative fit index (CFI) and the Tucker-Lewis index (TLI) should be > 0.95 (Schreiber et al., 2006). Bartlett’s test of sphericity and the Kaiser–Meyer–Olkin (KMO) test were performed to assess whether the data were suitable for factor analysis. Adequacy of the correlation matrix would be indicated by a significant Bartlett’s test (*p* < 0.05) and a KMO index > 0.70.

Measurement invariance refers to the generalisability element of construct validity (Putnick & Bornstein, 2016), and is assessed when scores need to be compared across groups (e.g. gender and language). Scales need to be invariant with respect to the way the latent constructs are formed (configural invariance), and the indicators or items should load similarly on latent factors across the groups (metric invariance). The requirement for invariance is that the difference in global χ*^2^* between hierarchical models is not significant. The measurement invariance for the EDS-S was evaluated for gender (men and women) and language (English first language speakers and non-English first language speakers).

Construct validity was explored by examining associations between the EDS-S and indicators of common mental disorders (PHQ-9, which was also coded for the presence of Major Depressive Disorder; GAD-7, also coded for the presence of Generalised Anxiety Disorder; PC-PTSD-5, also coded for the likelihood of PTSD; CAGE questionnaire, also coded for the likelihood of alcohol use disorder), as well as the other self-reported indicators of adjustment difficulties (as described earlier). Pearson’s correlations were calculated for scaled markers, while *t*-tests for independent samples were conducted for categorical markers (i.e. indicators with yes/no answers). Because ED has been associated with varying types of psychopathologies, divergence across psychiatric symptoms was not expected, and it was predicted that ED would show positive associations with mood, anxiety and alcohol use disorder symptoms, as well as psychiatric hospitalisations and lower global adaptive functioning (Powers et al., 2015, p. 86).

Positive findings of associations were explored further to determine the extent of each indicator’s contribution to variance on the EDS-S. A series of binomial logistic regressions were conducted for 12 indicators of common mental disorders and adjustment difficulties. Receiver operating/operator characteristics (ROC) curve analyses were also conducted for these indicators.

### Study 2

#### Overview of the study

The sample completed two instruments, in booklet form, in a cross-over design. One version of the booklet (‘Condition 1’) presented the questionnaires in the format of BRUMS first, then 10 affectively neutral biographical items and then the EDS-24. A second version of the booklet (‘Condition 2’) presented the questionnaires in the format of EDS-24 first, then 10 affectively neutral biographical items and then the BRUMS.

#### Participants and procedure

The sample consisted of naval administrative personnel who volunteered to complete the questionnaires during their biennial occupational health screen. The study booklet containing the scales was additional to their screening and was completed anonymously. Prior to the questionnaire administration, they were briefed that completion of the booklet would be considered as implied consent. Consequentially, no consent forms were completed, and the researchers could not know who had completed the booklet and who had not.

The sample of 168 had a mean age of 31.5 years (s.d. = 5.6, range: 21–50, with 65% concentrated in the 26–35-year age band), and included 25 (14.9%) women. All participants had at least a grade 12 education, with 88% also in possession of higher vocational training certificates. All self-identified as proficient in English. The two subgroups were well matched, with no significant differences in gender composition (χ^2^ < 0.001, *p* = 0.996) or mean age (*t* = 0.799, *p* = 0.426).

The full sample completed the questionnaire booklet in a single session. A total of 200 questionnaires were prepared (100 of each version) and were handed out randomly, resulting in the unequal subgroup sizes. Of the 174 booklets returned, six cases were excluded because of missing data points.

#### Measures

**Emotional Dysregulation Scale:** The 24-item EDS (Bradley et al., 2011) was administered, with full sample Cronbach α = 0.91.

**Brunel Mood Scale:** The BRUMS is a 24-item self-report inventory that measures transient affective mood states (Terry et al., 1999, 2003a), using a five-point Likert scale (0 = *not at all*, 4 = *extremely*). It has been used extensively, and a substantial body of literature exists on its use in many domains – from sports performance (Lane et al., 2005) to academic achievement (Thelwell et al., 2007), as well as a marker of mental health (Brandt et al., 2016). Good concurrent and criterion validity has been reported internationally (Terry et al., 1999, 2003a) and in SA (Terry et al., 2003b). A Cronbach α of 0.79 was calculated for this study. A total mood distress score – where higher scores represent greater distress – can be calculated and was used in this study.

#### Data analysis

The scales were administered in their standard format, and the respective total scores were calculated according to standard procedures. Only total scale scores are reported in this study. Scale associations were analysed using Pearson’s correlation coefficients. This was done for the total sample, as well as the two conditions. Priming effects were further explored with *t*-tests for independent samples (for both EDS-24 and EDS-S). Cohen’s *d* was employed to consider effect sizes. All statistical analyses were conducted using SPSS-27.

### Study 3

#### Overview of the study

A sample of SAN sailors preparing for a long-range maritime patrol (3-month duration) completed the EDS-S 1 week prior to departure. At the end of the mission, they completed a self-assessment of their performance relating to work-output, social relations and emotional stability during the patrol, and also completed the BRUMS.

#### Participants and procedure

The sample comprised 152 naval volunteers who consented to complete the scales and questionnaires immediately prior, and at completion of a ship-based operational patrol of 3 months. The sample had a mean age of 31.6 (±5.6, range: 21–50 years), and comprised 21 women (13.8%) and 131 men (86.2%). Of the total group, 76 (50%) worked in navy-specific fields, 49 (32.2%) in technical and engineering fields and 27 (17.8%) in support fields.

#### Measures

**Emotional Dysregulation Scale-Short Form:** The 12-item EDS-S (Powers et al., 2015) was administered, in English, in the week prior to departure. A mean total score = 15 (±5; range: 12–48) and Cronbach α = 0.89 were calculated for this sample.

**Brunel Mood Scale:** The 24-item BRUMS (Terry et al., 2003a) was administered, in English. This was done at week 12, at the end of the patrol. Cronbach α for this sample was 0.82.

**Self-report assessment of performance:** Participants were invited to rate their performance on the triarchy using a three-item, 10-point scale (1 = ‘*poor’*, 10 = *‘very good’*), with the instruction set referring to ‘during the past six weeks’. The items referred to: (1) ‘the quality of your work output’, (2) ‘the quality of your interpersonal interactions (e.g. how you got along with others)’ and (3) ‘the quality of your emotional state (e.g. how you were mostly feeling)’. This was done at week 12, at the same time as the BRUMS.

#### Data analysis

Pearson’s correlation coefficients were calculated, and linear regression analysis (with EDS-S as regressor) was used to predict both self-reported performance across the triarchy and mood state. All statistical analyses were conducted using SPSS-27.

### Study 4

#### Participants

Successful SAN submariners were invited to complete the EDS-S anonymously and briefed that completion of the scale will be considered as implied consent.

Submariners were considered successful (i.e. good adaptors) based on a number of criteria, including (1) completed at least 2 years of operational experience after qualification, (2) have no organisational record of poor psychological adaptation on submarines and (3) received positive supervisors’ reports, including a recommendation for continued use on-board submarines (personal correspondence, Institute for Maritime Medicine, 19 August 2022).

The sample of 48 participants had a mean age of 40.0 years (±6.9), comprised of 18 (37.5%) women and 30 (62.5%) men, with 18 (37.5%) reporting English as first language and 30 (62.5%) reporting other SA languages as their first language. English is the language spoken onboard the submarines. All participants were highly skilled and in possession of post-school tertiary academic training or advanced technical qualifications.

#### Measures and data analysis

The EDS-S was administered, in English. Descriptive statistics were calculated, as were differences between the sample’s mean score and that of the general worker sample reported in study 1, using a *t*-test for single samples.

### Ethical considerations

This project has been approved by the Health Research Ethics Committee of Stellenbosch University (reference number: N20/07/078).

## Results

### Study 1: Descriptive scale scores

The EDS-24 had a mean total score of 36.6 (±16.6) and a range of 24–152. Cronbach α = 0.93, and no deletion of items improved it. To confirm the 12-item EDS-S, the current study carefully replicated the original process undertaken by Powers et al. (2015). An EFA, using a scree-test, identified one factor (explaining 41.2% of variance) on which all items loaded. After the item-loadings were examined, the same 12 items were retained. As with the original study, a strong bivariate correlation was found between the 24-item and 12-item scales (*r* = 0.96, *p* < 0.001). The EDS-S had a mean total score of 17.6 (±8.7) and a range of 12–76. No floor or ceiling effects were detected.

### Study 1: Evidence for structural validity

**Socio-demographic effects:** Age showed a small but significant correlation to EDS-S scores (*r* = −0.171, *p* < 0.001). However, this was not a linear distribution, and an ANOVA (*F*_3,1002_ = 11.915, *p* < 0.001) indicated that higher scores were clustered in the age bracket 20–29 years. There were no significant differences in the mean total scores of women and men (*t* = -0.931, *p* = 0.352, Cohen’s *d* = 0.063), or of English first language and not-English first language speakers (*t* = 0.831, *p* = 0.4.7, Cohen’s *d* = 0.068).

**Internal consistency and test–retest reliability:** The EDS-S Cronbach α was 0.91, and no deletion of items improved it. Inter-item correlations ranged from 0.323 to 0.600, while corrected item-total correlations ranged from 0.557 to 0.742. The EDS-S showed good temporal stability over 35 days (*t* = 1.1914, *p* = 0.07; mean difference = 0.6; *r* = 0.950, *p* < 0.001).

**Dimensionality:** The correlation matrix was adequate for factor analysis (Bartlett’s test = 5540.852; degree of freedom [*df*] = 66; *p* < 0.001; KMO = 0.942), and the 12-item EDS-S was subjected to CFA. Although the one-factor model did not obtain a non-significant χ^2^ (χ^2^ = 379.118, *df* = 54), the value was not excessively high. Further, while not an absolute fit, the RMSEA (0.067; 90% CI: 0.061–0.076) was adequately small (< 0.08), and the CFI (0.941) and TLI (0.928) also supported an adequate fit. Standardised loadings were relatively uniform, ranging from 0.59 to 0.79. Thus, the unidimensional model appeared to have an acceptable fit to the data.

**Measurement invariance:** The EDS-S for women and men showed acceptable configural invariance but did not reach metric invariance (Δχ^2^ = 79.68, Δ*df* = 11, *p* < 0.001). The EDS-S for English first language speakers and non-English first language speakers also showed acceptable configural invariance but again did not reach metric invariance (Δχ^2^ = 37.40, Δ*df* = 11, *p* < 0.001).

### Study 1: Evidence for construct validity

Correlations between the EDS-S and screeners for common mental disorders were all significant (*p* < 0.001). Emotional dysregulation correlated significantly and positively with clinical measures of depression (PHQ-9, *r* = 0.540) and general anxiety (GAD-7, *r* = 0.540), with large effect sizes. Significant positive correlations were also observed for PTSD (PC-PTSD-5, *r* = 0.372) and stress overload (SOS-S, *r* = 0.496), with moderate effect sizes. The positive correlation with the measure of problematic alcohol use was significant (CAGE, *r* = 0.264) but with small effect size.

Emotional Dysregulation Scale-Short Form total scores further differentiated significantly between individuals with positive indicators on all the mental health and adjustment difficulty questions, and those without (*p* > 0.001), which are presented in Table 3. Large effect sizes (Cohen’s *d* ≥ 0.8) were observed for all 12 indicators.

**TABLE 3:** *T*-test for independent samples for Emotional Dysregulation Scale-Short Form and selected indictors of common mental disorders and other adjustment difficulties.

Indicator	NO			YES			*t*	*p*	Cohen’s *d*
	*n*	Mean	s.d.	*n*	Mean	s.d.			
Major depressive disorder	963	17.3	8.1	20	36.3	16.0	−10.046	< 0.001	2.27
Generalised anxiety disorder	993	17.3	8.2	13	38.8	16.4	−9.196	< 0.001	2.57
Post-traumatic stress disorder	993	17.4	8.2	13	35.2	21.7	−7.562	< 0.001	2.11
Alcohol use disorder	997	17.4	8.4	9	36.0	19.1	−6.507	< 0.001	2.18
Adult attention-deficit/hyperactivity disorder	889	16.4	7.1	110	27.1	13.6	−13.161	< 0.001	1.33
Previous psychiatric admission	921	17.4	8.3	18	30.0	19.5	−6.094	< 0.001	1.45
Previous out-patient psychological/psychiatric treatment	887	17.1	7.9	53	27.4	15.5	−8.544	< 0.001	1.21
Previous treatment for alcohol or substance abuse or addiction	932	17.5	8.6	9	30.3	20.0	−4.364	< 0.001	1.46
Interpersonal difficulties in a workgroup (conflict with co-workers, supervisors)	904	17.4	8.5	36	24.6	12.8	−4.884	< 0.001	0.83
Disciplinary issues at work (past 2 years)	919	17.5	8.7	22	24.4	13.0	−3.648	< 0.001	0.79
Domestic discord	968	17.1	7.7	38	31.7	17.2	−10.774	< 0.001	1.78
Stress overload	195	16.2	5.8	29	26.7	11.0	−7.961	< 0.001	1.58

s.d., standard deviation.

The results of the binomial logistic regressions, as well as the results of the ROC curve analysis, are presented in Table 4. The binomial logistic regressions for all 12 indicators of common mental disorders and adjustment difficulties were statistically significant (*p* < 0.01). The model for each indicator of common mental disorders explained 19% – 29% of variance. The model for each indicator of life difficulties explained 5% – 20% of variance. The logistic regression further correctly classified 87.5% – 99.1% of cases. Clinically useful (> 80%) areas under the curve were reported for mental disorders, and acceptable areas under the curve were reported for other indicators of more general adjustment difficulties (66% – 77%).

**TABLE 4:** Binomial regression predicting selected indicators of common mental disorders and other adjustment difficulties using Emotional Dysregulation Scale-Short Form scores.

Indicator	Nagelkerke *R*^2^†	*χ* ^2^	PAC	Wald	OR	95% CI	AUC
Major depressive disorder	24	43.430**	97.9	45.577**	1.10	1.07–1.14	0.874
Generalised anxiety disorder	26	33.821**	98.7	36.671**	1.10	1.07–1.14	0.920
Post-traumatic stress disorder	19	25.029**	98.9	30.308**	1.09	1.06–1.13	0.835
Alcohol use disorder	18	18.163**	99.1	23.257**	1.09	1.05–1.13	0.871
Adult attention-deficit/hyperactivity disorder	20	103.898**	89.2	90.033**	1.10	1.08–1.12	0.803
Previous psychiatric admission	12	19.337**	98.1	24.663**	1.08	1.05–1.11	0.721
Previous out-patient psychological/psychiatric treatment	13	42.636**	94.7	46.243**	1.08	1.05–1.10	0.752
Previous treatment for alcohol or substance abuse or addiction	10	9.769*	99.0	13.624**	1.07	1.03–1.11	0.685
Interpersonal difficulties in a workgroup (conflict with co-workers, supervisors)	6	15.458**	96.2	19.234**	1.06	1.03–1.08	0.714
Disciplinary issues at work (past 2 years)	5	8.648*	97.7	11.329*	1.05	1.02–1.08	0.669
Domestic discord	20	56.391**	96.2	58.006**	1.10	1.07–1.12	0.774
Stress overload	29	38.484**	87.5	29.965**	1.16	1.10–1.22	0.795

PAC, percentage accuracy in classification; OR, odds ratio; 95% CI, 95% confidence interval; AUC, area under the curve.

* , *p* < 0.01;

** , *p* < 0.001.

† , % variance explained.

### Study 2: Priming effect

The BRUMS total mood distress score for the full sample was -7.75 (±6.6, range: -16 to 16), the mean total score for the EDS-24 was 32.42 (±10.9; range: 24–88) and 15.5 (±5.3; range: 12–48) for the EDS-S. The full sample scale totals for the BRUMS and EDS-24 correlated significantly and positively (*r* = 0.502, *p* < 0.001). Stronger correlations were found for the Condition 1 sample (*r* = 0.610, *p* < 0.001) than for the Condition 2 sample (*r* = 0.438, *p* < 0.001).

The scale total score outcomes of the *t*-tests for independent samples are reported in Table 5. Three individual items of the EDS-24 represented the largest (0.4–0.6) mean differences.

**TABLE 5:** The outcome of the *t*-tests for independent samples for Brunel Mood Scale and Emotional Dysregulation Scale.

Instrument	Condition 1			Condition 2			*t*	*p*	Cohen’s *d*	Mean difference
	*n*	Mean	s.d.	*n*	Mean	s.d.				
BRUMS	74	−7.85	6.7	94	−7.67	6.4	−0.177	0.860	6.6	0.2
EDS-24	74	35.68	12.7	94	29.86	8.6	3.369	0.001	11.9	5.8
EDS-S	74	16.74	6.4	94	14.46	3.9	2.847	0.005	5.2	2.3
Age	74	31.9	6.3	94	31.2	5.0	0.799	0.426	5.6	0.7

s.d., standard deviations; BRUMS, Brunel Mood Scale; EDS-S, Emotional Dysregulation Scale-Short Form.

### Study 3: Prediction of performance in isolated, confined and extreme contexts

The correlations between EDS-S scores and self-report performance and mood state are presented in Table 6. Baseline ED correlated significantly to both self-rated performance and self-report mood state, with modest effect sizes. Table 7 presents outcomes of a linear regression analysis, where the EDS-S significantly predicted self-rated performance and mood state during an ICE environment exposure, again with modest effect sizes.

**TABLE 6:** Correlations between baseline Emotional Dysregulation Scale-Short Form scores and self-rated performance at end of deployment (week 12).

Measure	EDS-S	
	*r*	*p*
**At week 12 (*n* = 152)**	
Quality of work output	−0.178	0.030
Quality of interpersonal interactions	−0.331	0.000
Quality of emotional state	−0.336	0.000
BRUMS	0.293	0.000

BRUMS, Brunel Mood Scale; EDS-S, Emotional Dysregulation Scale-Short Form.

**TABLE 7:** Linear regression analysis with Emotional Dysregulation Scale-Short Form.

Performance rating	*F*	Beta	*t*	*p*
Quality of work output	4.732	0.176	−2.175	0.031
Quality of social interaction	18.328	0.332	−4.278	< 0.001
Quality of emotional state	18.329	0.332	−4.281	< 0.001
BRUMS	14.120	0.293	3.758	< 0.001

BRUMS, Brunel Mood Scale.

### Study 4: Emotional Dysregulation Scale-Short Form profile of SAN submariners

The submariners had a mean EDS-S score of 12.9 (±1.2, range: 12–16). There were no significant differences between the mean scores of English first language and non-English first language speakers (*t* = -1.848, *p* = 0.07), and the sample mean was significantly lower than that of the general worker sample (*t* = -25.534, *p* < 0.001, Cohen’s *d* = 3.69).

## Discussion

The mean EDS-S total score of the current (non-clinical) workplace sample was substantially lower than earlier studies that focussed on vulnerable individuals (e.g. with history of psychological trauma, psychiatric disorders, etc.). The degree of difference could in part be attributed to the fact that the current sample consisted of generally healthy and employed individuals who had access to employer-sponsored health and well-being services. Cronbach’s α was similar to published studies.

### Evidence of validity

The first aim was to explore evidence of structural validity. The lack of significant gender effects was expected (Powers et al., 2015), and age effects were consistent with previous reports that suggested that, as people get older, they learn to cope with stressors and avoid emotionally triggering situations (Raimondi et al., 2022, p. 424).

Evidence for structural validity could be found in the acceptable unidimensional model fit, similar to the Italian version (Raimondi et al., 2022) in this non-clinical population. Good internal reliability and temporal stability (at least over the short term) were also demonstrated. However, the EDS-S only achieved configural measurement invariance, but not metric invariance, for both gender and language. In the absence of significant differences in mean total scores between gender and language groups, this finding would require further exploration. Thus, evidence of structural validity was found, although the limited measurement invariance suggests the need for some caution in practical application.

The second aim was to explore evidence of construct validity. In this regard, significant associations with measures of psychopathology and general adjustment were demonstrated. Emotional Dysregulation Scale-Short Form total mean scores were associated with indicators of mood, anxiety, problematic alcohol use, PTSD, ADHD, and history of psychiatric hospitalisations and mental health treatment. Emotional Dysregulation Scale-Short Form total mean scores were also associated with indicators of general adjustment difficulties, including problematic interactions in the workplace and at home, and could differentiate between individuals with positive indicators on all the markers of mental health and adjustment difficulties, and those without. The EDS-S appeared particularly useful in predicting depression, general anxiety and stress overload.

Previous reports established the association of ED with varying types of psychopathologies, and thus divergence across psychiatric symptoms was not expected in this study. Indeed, in support of earlier findings (Christ et al., 2019; Mandavia et al., 2016; Mekawi et al., 2020; Michopoulos et al., 2015; Pencea et al., 2020; Powers et al., 2015; Raimondi et al., 2022), the EDS-S predicted symptoms and indicators associated with mood and anxiety disorders, PTSD, history of psychiatric hospitalisation and problematic substance use, as well as non-clinical indicators of adjustment difficulties. This finding supports the understanding of ED as a trans-diagnostic process that impacts many psychological conditions and experiences, both clinical disorders and non-clinical indicators of adjustment.

Further, low and homogenous EDS-S scores were found in an ICE sample expected to have homogenously low scores, namely navy submarine crews, a group of proven good adaptors in their ICE context. Their low scores, consistently observed throughout the sample, suggest low ED and good adaptation.

Thus, evidence of construct validity were demonstrated, in these non-clinical samples of general workers and SAN specialists.

### Consideration for practical use

**Priming:** The two conditions in study 2 were equal in age, gender and BRUMS scores, but significantly different in EDS scores, depending on the order of administration. When the BRUMS was completed first, there were significantly higher ED scores than when the EDS was completed first (mean difference 5.8, representing half a s.d. from the full sample mean). Three EDS-24 items had particularly large mean differences between conditions (> 0.4), and might be particularly susceptible to priming. In this regard, it is noteworthy that these three items have already been removed in the EDS-S, and that the mean difference between EDS-S total scores across conditions represent less than half a SD from the full sample mean, suggesting that the short form might be more resilient to priming.

Prior completion of the EDS did not appear to bias responses to the BRUMS. The EDS asks questions in a ‘general’ sense, which may make it more susceptible to priming, whereas the BRUMS asks about specific current timeframes, thus possibly offering less opportunity for priming.

In summary, the scale was found to be potentially vulnerable to priming bias, which may need to be considered when it is included in battery format administration. It may be particularly susceptible to the effects of measures with very specific instruction frames when sequenced prior to EDS administration. The short form appeared more resilient to priming effects, suggesting its preferential use (as opposed to the 24-item version) in battery administration.

**Prediction of performance:** The EDS-S predicted self-rated performance in this sample deployed into an ICE context. However, the effect sizes were very modest, which could limit its value for practical application in this context at this time. The smaller effect sizes may in part be because of the practise that all navy sailors undergo an annual mental health screening, and only those without debilitating mental health concerns would then be eligible for deployment. This was visible in the current sample, in the limited range of scores on the EDS-S, and in that no ED was noted in participants’ responses. It could also be hypothesised that the relatively short time frame of 3 months may not be enough to elicit more severe expression of ED. Sailors might be able to cope over short periods, whereas maintaining good emotional regulation may become more difficult over longer time frames. The fact that both the ED and rated performances were self-reported, was a limitation to this study, and more research may be required to confirm its practical utility in ICE environments.

### Practical application

This study’s findings have immediate practical application to ICE workplaces: Across varying iterations, ICE environments place greater demands on individuals’ and groups’ adaptive functioning capacities than is typically found in more conventional environments (Palinkas & Suedfeld, 2008; Sandal, 2000; Shea et al., 2009). Isolation and confinement also decrease an individual’s ability to regulate emotions (Liu et al., 2016), making people in ICE settings vulnerable to health problems, reduced emotional well-being, decreased performance and interpersonal tension (Basner et al., 2014; Palinkas & Suedfeld, 2008; Sandal, 2000; Shea et al., 2009). When included as part of a comprehensive psychological assessment, the EDS-S could become a useful tool to assess risk for poor emotional regulation, serving three purposes: (1) Assessment of ED risk could guide the selecting-out of individuals with high-risk profiles; (2) knowing risk profiles could allow for increased support through closer monitoring of high-risk individuals, either by remote programme directors, or local expedition medical staff and (3) the EDS-S could be used to better prepare individuals – prior to ICE missions – through greater awareness of their own ED risks and the development of coping strategies to enhance appropriate emotional self-regulation. Such initiatives could be employed by the SAN on their ships and submarines or in other military missions (e.g. current protracted peacekeeping operations across Africa), as well as the South African National Antarctic Programme. This may also be useful for private companies in the offshore oil and gas industry for the selection, preparation and placement of staff.

The association of ED with adverse childhood experiences (ACE) has previously been demonstrated by the EDS (Bradley et al., 2011; Christ et al., 2019; Mandavia et al., 2016). South Africa has many young adults with history of ACE (Manyema & Richter, 2019), and possibly even more children currently experiencing ACEs, which may warn of the risk of a major mental health epidemic in the near future. With the current evidence of validity, the EDS-S can now be used with some confidence in local studies of similar populations, and particularly with investigations into the association of ACE, ED as adults, and associated poor mental health and adjustment outcomes. The EDS-S can further be used in mental health service settings to guide targeted treatment for persons with depressive or anxiety symptoms (Fehlinger et al., 2013; Mennin, 2006).

### Limitations and future directions

The study used non-clinical samples of workplace populations who were generally well educated and in good health, with good self-reported English proficiency. Results cannot necessarily be generalised to the wider SA population, and additional samples from diverse sectors of society would be helpful to confirm the results. Further, assessment tools like the EDS-S rely on respondents’ literacy with regard to the semantic descriptions of emotional distress. Individuals without the English proficiency of the current samples might be challenged to express their experience of emotional regulation in English. Future research would be invaluable to validate the EDS-S in samples with different levels of language proficiency. Future studies also need to test this instrument in clinical samples and other groups vulnerable to poor mental health outcomes. Further exploration of measurement invariance, across gender and language, would provide further confidence in the EDS-S. Finally, future studies need to test the application of the EDS-S across different ICE contexts (e.g. ships at sea vs weather stations on isolated islands), across different time frames (shorter vs longer missions) and to use more objective ratings of performance across the triarchy (e.g. supervisor or peer rating of quality of work and interpersonal relations, and extended measures for emotional well-being).

## Conclusion

This study reported evidence of validity for the 12-item EDS-S. It made a novel contribution in that it replicated previous investigations in a SA context: evidence of structural and construct validity was demonstrated, in non-clinical samples of SA workers, and significant associations with measures of mental health and adjustment difficulties were reported. This study further provided preliminary support for the EDS-S to predict self-rated performance in ICE environments.

There is some support for the use of the scale in clinical research (e.g. exploring associations between ED and ACE) and applied practise (e.g. assessment of psychological performance in ICE environments). However, caution must be observed for possible effects of language proficiency, and further research into the role of language is required.
